# Emotionales Erleben von Schüler*innen in Jahrgangsstufe 4 unter dem Einfluss der Covid-19-Pandemie im Schuljahr 2019/2020

**DOI:** 10.1007/s42278-022-00151-0

**Published:** 2022-06-20

**Authors:** Ulrike Nett, Sonja Ertl, Tanja Bross

**Affiliations:** 1 Empirische Bildungsforschung, Philosophisch-Sozialwissenschaftliche Fakultät, Universität Augsburg, Universitätsstraße 10, 86159 Augsburg, Deutschland; 2 Institut für Grundschulforschung, Friedrich-Alexander-Universität Erlangen-Nürnberg, Regensburger Str. 160, 90478 Nürnberg, Deutschland

**Keywords:** Emotionen, Emotionales Erleben, Covid-19-Pandemie, Distanzlernen, Übertritt, Emotions, Emotional experience, Covid-19 pandemic, Distance learning, Transition

## Abstract

Bisherige empirische Befunde belegen, dass Lern- und Leistungsemotionen in Wechselwirkung mit Schulleistungen stehen und von situativen und individuellen Faktoren beeinflusst werden. In Jahrgangsstufe 4 ist dabei vor allem der nahende Übertritt in eine weiterführende Schule mit den damit verbundenen schuljahresspezifischen Ereignissen, beispielsweise der Zwischeninformation oder dem Übertrittszeugnis, von Bedeutung – im Schuljahr 2019/2020 kam der Covid-19-bedingte Lockdown mit dem damit verbundenen Distanzlernen hinzu. Im Beitrag wird der Frage nachgegangen, wie sich das emotionale Erleben vom Ende der 3. bis zum Ende der 4. Jahrgangsstufe in Abhängigkeit schuljahresspezifischer sowie individueller Faktoren entwickelt. Dazu wurden in einer längsschnittlichen Fragebogenstudie 225 Schüler*innen in Bayern zu ihren fachbezogenen Emotionen sowie bzgl. des bevorstehenden Übertritts befragt. Es zeigt sich, dass die Schüler*innen insgesamt eher günstige Emotionen erleben. Das Distanzlernen geht mit signifikanten Veränderungen im emotionalen Erleben einher. Hinsichtlich individueller Einflussfaktoren erweisen sich insbesondere die Schulleistung, teilweise auch Geschlecht und Migrationshintergrund, als bedeutsam für das emotionale Erleben. Die Ergebnisse werden diskutiert und weitere Forschungsdesiderata aufgezeigt.

## Abstract (en)

Recent empirical studies support the interaction between achievement emotions with school performance. Situational as well as individual factors were found to have an impact on these emotions. In grade 4, the approaching transition to secondary school is a particularly important event and is associated with specific further events, such as school reports. During the year 2019/2020, the Covid-19-induced lockdown, in Germany associated with distance learning, was also an event that might be of impact. The present paper explores the question of how emotional experiences develop from the end of 3rd to the end of 4th grade as a function of school-year-specific as well as individual factors. In a longitudinal questionnaire study, 225 students in Bavaria rated their subject-related emotions and their emotions towards the upcoming transition. In general the results show that the students experience a positive pattern of emotions. The distance learning is accompanied by significant changes in students’ emotional experiences. School performance and, to some extend the individual factors gender and migration background, have a significant impact on the emotional experiences as well as changes in these emotional experiences. The results are discussed and further research desiderata are identified.

## Problemaufriss

Emotionen sind im schulischen Kontext sowohl für die Persönlichkeitsentwicklung als auch für das Lernen und Leisten der Schüler*innen bedeutsam (Frenzel und Goetz 2018). Die Lern- und Leistungsemotionen stehen dabei in reziproker Wechselwirkung mit der Schulleistung (z. B. Pekrun et al. 2017). Erklären lässt sich dies mit der Kontroll-Wert-Theorie (z. B. Pekrun et al. 2006), die auf der Appraisaltheorie basiert: Je nachdem, in welchem Umfang und welcher Intensität man glaubt, Situationen oder auch Handlungsergebnisse kontrollieren zu können und diese wertschätzt, werden diese positiv oder negativ bewertet. Diese Bewertungen (appraisals) führen dann zu eher positiven oder negativen Emotionen.

Der Eintritt in die vierte Jahrgangsstufe stellt für viele Schüler*innen der vierjährigen Grundschule einen besonderen Meilenstein dar. Während der vierten Jahrgangsstufe rücken die Schulleistungen aufgrund damit verbundener Empfehlungen oder Berechtigungen für die weiterführende Schule verstärkt in den Vordergrund. Im Schuljahr 2019/2020 kam das durch die Covid-19-Pandemie bedingte Distanzlernen als besonderes Ereignis hinzu.

Es gibt bislang kaum Studien, die sich auf Lern- und Leistungsemotionen von Schüler*innen im Grundschulalter beziehen. Es liegen zwar Ergebnisse zum emotionalen Erleben des Übertritts vor (z. B. Hartinger et al. 2004; Kurtz et al. 2010), zum emotionalen Erleben der Lernenden in dieser Zeit, unter Berücksichtigung schuljahresspezifischer Ereignisse (z. B. dem Erhalt eines Übertrittszeugnisses), sind uns bislang keine Studien bekannt. Auch sind uns keine Studien bekannt, die das emotionale Erleben in Jahrgangsstufe 4 unter dem Einfluss des Covid-19-bedingten Distanzlernens untersuchten. In der vorliegenden Studie soll daher der Frage nachgegangen werden, wie sich das emotionale Erleben der Lernenden vom Ende der Jahrgangsstufe 3 bis zum Ende der Jahrgangsstufe 4 – unter dem Einfluss des ersten Covid-19-bedingten Distanzlernens – entwickelt.

## Emotionales Erleben

Lern- und Leistungsemotionen wurden lange Zeit als sogenannte stabile Konstrukte erfasst (z. B. Lichtenfeld et al. 2012; Pekrun et al. 2011). Ein elementares Merkmal von Emotionen ist jedoch ihre Eigenschaft, dass sie nicht stabil sind, sondern sich permanent und situationsabhängig verändern (Frijda 2007), wie auch in der Kontroll-Wert-Theorie selbst dargestellt (Pekrun et al. 2006). Erst in jüngster Zeit wurde diese Tatsache mit Studien, die emotionales Erleben von Schüler*innen auch in spezifischen Situationen erfassen, berücksichtigt (z. B. Nett et al. 2017; Goetz et al. 2020). Allerdings wurden diese Studien bisher vor allem mit älteren Schüler*innen durchgeführt. Für die Altersstufe der Grundschüler*innen konnte Helmke (1993) ein Absinken der Lernfreude während der Grundschulzeit belegen. Reindl und Hascher (2013) zeigten – mit einer allerdings vergleichsweisen kleinen Stichprobe – für das Fach Mathematik, dass in allen Jahrgangsstufen die positiven Emotionen höher ausgeprägt sind als die negativen. Zudem ist ein „Rückgang positiver Emotionen und damit […] eine zunehmend distanzierte Haltung gegenüber Mathematik“ (ebd., S. 268) von der 1. bis zur 4. Jahrgangsstufe zu verzeichnen. Des Weiteren konnten sie in ihrer Studie einen Erholungseffekt im Bereich der negativen Emotionen über die Sommerferien feststellen. Insgesamt legen die Befunde nahe, dass spezifische Situationen einen Einfluss auf das emotionale Erleben von Schüler*innen haben und somit im Verlauf der ereignisreichen 4. Jahrgangsstufe im Schuljahr 2019/2020 auch von Veränderungen im emotionalen Erleben der Schüler*innen auszugehen ist.

### Emotionales Erleben und die Herausforderungen in Jahrgangsstufe 4

Zu den situativen Einflussfaktoren zählt in Jahrgangsstufe 4 insbesondere der anstehende Übertritt auf eine weiterführende Schule und damit verbundene Leistungsrückmeldungen. Der Übertritt von der Grundschule in die weiterführende Schule stellt aufgrund seines festgelegten Zeitpunktes und der Gültigkeit für alle Kinder ein normatives Ereignis (Kurtz et al. 2010) und damit einen normativen Stressor dar (Beyer und Lohaus 2007, S. 12). Neben dem eigentlichen Übertritt sind in Bundesländern mit Übertrittszeugnis (z. B. Bayern, in dem die vorliegende Studie durchgeführt wurde) weitere möglicherweise stress- und emotionsgeladene Zeitpunkte im Laufe des vierten Schuljahres vorhanden. So erhalten die Lernenden im Januar des Übertrittsjahres eine sogenannte Zwischeninformation, die den aktuellen Leistungsstand bezogen auf den nahenden Übertritt wiederspiegelt und im Mai schließlich das Übertrittszeugnis als „Berechtigung“ für bestimmte Schularten. Der Notenschnitt in den übertrittsrelevanten Fächern Deutsch, Mathematik und Heimat- und Sachunterricht „berechtigt“ für die unterschiedlichen weiterführenden Schularten (ohne einen Probeunterricht erfolgreich absolvieren zu müssen). In Bundesländern mit Übertrittsempfehlung ist diese nicht bindend und die Wahl der weiterführenden Schulart obliegt den Erziehungsberechtigten (und ihren Kindern). Der Übertritt wird zwar von den meisten Kindern eher als Herausforderung anstatt als Bedrohung wahrgenommen (Kurtz et al. 2010), es zeigen sich aber Einflüsse schuljahresspezifischer Ereignisse, wie bspw. dem Übertrittszeugnis. In einer Untersuchung von Reinders et al. (2014) zeigte sich eine höhere Stressbelastung bei Kindern, welche ein verbindliches Übertrittszeugnis erhalten haben, im Vergleich zu Kindern, die „nur“ eine Übertrittsempfehlung erhalten haben. Die höchsten Stresswerte wiesen dabei Kinder an der Notenschwelle zwischen Mittel- (früher: Hauptschule) und Realschulempfehlung auf, mit einem dramatischen Anstieg von der dritten zur vierten Klasse (ebd., S. 4). In einer weiteren Untersuchung zeigten Schüler*innen, bei denen ein Übertritt an ein Gymnasium oder eine Realschule aufgrund des Notenschnitts in den übertrittsrelevanten Fächern nicht gesichert, aber möglich ist, deutlich höhere Werte bezüglich der Leistungsangst als leistungsstarke Schüler*innen (Hartinger et al. 2004, S. 186).

Im Schuljahr 2019/2020 stellte darüber hinaus das Covid-19-bedingte Distanzlernen ein weiteres herausforderndes Ereignis dar. Nach Aussage der Eltern haben bei 25 % der Kinder durch die Covid-19-Pandemie emotionale Probleme zugenommen (Wößmann et al. 2021, S. 45). Auch die Copsy-Studie berichtet von einer Zunahme der Belastung durch die Pandemie, auch im Bereich des emotionalen Erlebens (Ravens-Sieberer et al. 2021). Zudem kann sich auch die veränderte Unterrichtssituation auf das emotionale Erleben – positiv wie negativ – auswirken. So bietet das individuelle häusliche Lernen mehr Möglichkeiten des Autonomieerlebens und gleichzeitig weniger Möglichkeiten des Kompetenzerlebens und der sozialen Eingebundenheit, da im Distanzlernen ca. in 63 % der Fälle die Grundschulkinder nur einmal, weniger als einmal in der Woche oder nie eine Rückmeldung zu den bearbeiteten Aufgaben erhielten und bei fast der Hälfte der Kinder während der Schulschließungen 2020 kein gemeinsamer Unterricht für die ganze Klasse (z. B. per Videoanruf) stattgefunden hat (Wößmann et al. 2020, S. 33).

Ein weiterer, mit der Situationsspezifität des emotionalen Erlebens von Schüler*innen verbundener, Aspekt ist die Domänenspezifität von Lern- und Leistungsemotionen. Für den Sekundarschulbereich konnte die Domänenspezifität von Emotionen bereits mehrfach belegt werden (vgl. Goetz et al. 2013b; Gogol et al. 2016). Im Grundschulbereich zeigen Studien von Lichtenfeld et al. (2012) sowie von Raccanello et al. (2013, 2019), dass bereits jüngere Schüler*innen in ihren unterrichtsbezogenen Emotionen zwischen Fächern bzw. Inhalten unterscheiden. Somit sollten schulspezifische Emotionen in Bezug auf einen fachlichen Fokus erfasst werden.

### Emotionales Erleben und Schulleistung

Zahlreiche Studien haben inzwischen den Zusammenhang zwischen Emotionen und akademischer Leistung vorrangig in der Sekundarstufe (z. B. Pekrun et al. 2017) oder an der Hochschule (z. B. Respondek et al. 2019) in den Blick genommen. Einen Überblick über die Studien und insbesondere den Zusammenhang von diskreten Emotionen und Leistung gibt die Metaanalyse von Camacho-Morles et al. (2021). Relativ wenig ist bisher über das emotionale Erleben von Grundschüler*innen in Zusammenhang mit Schulleistung bekannt (Ausnahmen z. B. Helmke 1993; Lichtenfeld et al. 2012; Raccanello et al. 2019).

Für das Fach Mathematik belegte Helmke bereits 1993 in einer längsschnittlichen Untersuchung im Primarschulbereich den Zusammenhang zwischen Lernfreude und Leistung. Dabei zeigte sich, dass der Effekt von Leistung auf Lernfreude stärker ausfällt als derjenige von Lernfreude auf Leistung.

In der internationalen Studie von Raccanello et al. (2019) zeigt sich für die Emotionen Freude, Angst und Langeweile – untersucht im muttersprachlichen Unterricht im entsprechenden Land und im Fach Mathematik – dass die Lern- und Leistungsfreude in Mathematik positiv mit der Leistung zusammenhängt. Für den muttersprachlichen Unterricht konnte dies nicht bestätigt werden. Für die Leistungsangst konnten negative Zusammenhänge mit der Leistung in beiden Unterrichtsfächern bestätigt werden. Für die Emotion Langeweile zeigt sich ein negativer Zusammenhang mit Leistung in Mathematik, aber nicht im muttersprachlichen Unterricht. Die Ergebnisse stehen in Einklang mit den Befunden von Lichtenfeld et al. (2012) für das Fach Mathematik. Krinzinger et al. (2009) konnten dagegen keinen Zusammenhang zwischen Leistungsangst und den mathematischen Fähigkeiten feststellen.

Möglicherweise sind die teils inkonsistenten Zusammenhänge zwischen Lern- und Leistungsemotionen und Leistung dadurch bedingt, dass sich im Grundschulalter insbesondere die Appraisals Kontrolle und Wert noch verändern (i. d. R. absinken; z. B. Jacobs et al. 2002; Wigfield et al. 1997). Entsprechend der Kontroll-Wert-Theorie sollten sich diese Veränderungen im subjektiven Erleben von Kontrolle und Wert auch auf das emotionale Erleben von Schüler*innen auswirken.

### Emotionales Erleben im Zusammenhang mit Geschlecht und Migrationshintergrund

Individuellen Einflussfaktoren, wie beispielsweise Geschlecht oder Migrationshintergrund, kommt in Bezug auf Lern- und Leistungsemotionen eine besondere Rolle zu.

Bezüglich des Geschlechts zeigen sich beispielsweise für das Fach Mathematik günstigere emotionale Muster für Jungen als für Mädchen (z. B. Frenzel et al. 2007; Helmke 1993; Lichtenfeld und Stupnisky 2013). Allerdings werden diese Unterschiede teilweise durch Facetten der Selbstwahrnehmung, wie beispielsweise dem Selbstkonzept, mediiert (Bieg et al. 2014; Goetz et al. 2013a). Lichtenfeld und Stupnisky (2013) fanden darüber hinaus Hinweise, dass sich Geschlechterunterschiede im Laufe der Zeit verstärken. Dass die individuellen Einflussfaktoren nicht nur einen Einfluss auf das emotionale Erleben insgesamt haben, sondern auch im Zusammenhang mit spezifischen situativen Einflussfaktoren stehen, wird auch in den Befunden zum Erleben des Übertritts als Herausforderung und Bedrohung deutlich (z. B. Kurtz et al. 2010). So wird der Übertritt, wie bereits erwähnt, von den meisten Kindern – sowohl im Bereich der Leistungen als auch im Bereich des Sozialen – eher als Herausforderung und weniger als Bedrohung wahrgenommen, allerdings in Abhängigkeit von individuellen Merkmalen der Schüler*innen (ebd.): Es zeigte sich, dass die Jungen im Vergleich zu den Mädchen mehr Bedrohung erleben. Darüber hinaus ergab sich, dass das Bedrohungserleben mit „einer höheren sozioökonomischen Stellung, einem hohen beruflichen Bildungsabschluss der Eltern, keinem Migrationshintergrund, höheren Schulleistungen, besseren Schulnoten, einer Gymnasialempfehlung sowie einer höheren wahrgenommenen Wertschätzung durch die Eltern (Responsivität) sinkt“ (ebd., S. 343).

Mit Bezug auf den Übertritt zeigt auch Hildebrandt (2014), dass Schüler*innen mit türkischem Migrationshintergrund ungünstigere Emotionen erleben als Schüler*innen ohne Migrationshintergrund. Allerdings konnten auch hier verstärkende (schlechte Schulleistung) und abmildernde (schulisches Selbstvertrauen) Faktoren identifiziert werden. Tobisch et al. (2016) konnten für den Primarbereich belegen, dass Schüler*innen mit Migrationshintergrund mehr Leistungsangst und verstärkte Hilflosigkeit erleben als Schüler*innen ohne Migrationshintergrund.

Obwohl die Befundlage zu den individuellen Einflussfaktoren Geschlecht und Migrationshintergrund bisher noch sehr unsicher ist, geben bisherige Studien erste Hinweise auf ihre Bedeutung. Aus diesem Grund erscheint es uns wichtig, diese Einflussfaktoren bei der Analyse des emotionalen Erlebens von Schüler*innen in Jahrgangsstufe 4 zu berücksichtigen.

## Forschungsfragen

Im vorliegenden Beitrag wird folgenden Fragestellungen nachgegangen.

I. Wie verändert sich das emotionale Erleben von Schüler*innen in Bezug auf Fachunterricht und Übertritt vom Ende der 3. Jahrgangsstufe bis zum Ende der 4. Jahrgangsstufe unter der Bedingung des Covid-19-bedingten Distanzlernens?

Zur Untersuchung dieser Fragestellung sollen besondere Zeitpunkte im Schuljahr, der Wechsel von der 3. in die 4. Jahrgangsstufe, der Erhalt der Zwischeninformation und das Covid-19-bedingte Distanzlernen in den Blick genommen werden. Dabei soll nicht nur ein allgemeiner Trend in den Veränderungen des emotionalen Erlebens, sondern auch die Varianz im emotionalen Erleben zwischen den Schüler*innen betrachtet werden. Da es sich beim Übertritt auf die weiterführenden Schulen um einen normativen Stressor handeln kann (s. oben), wird erwartet, dass damit verbundene Ereignisse mit einer Veränderung des emotionalen Erlebens der Schüler*innen in Zusammenhang stehen können. Auch das Distanzlernen während dieses Schuljahres könnte – aufgrund der Zunahme emotionaler Belastungen während der Covid-19-Pandemie (Ravens-Sieberer et al. 2021; Wößmann et al. 2021), und des damit veränderten Lernens (Wößmann et al. 2020) mit Veränderungen im emotionalen Erleben einhergehen.

II. In welchem Zusammenhang stehen Schulleistung, Geschlecht und Migrationshintergrund mit dem emotionalen Erleben der Schüler*innen in Jahrgangsstufe 4?

Aufgrund der Befunde von Kurtz et al. (2010) zum Erleben des Übertritts als Herausforderung oder Bedrohung wird ein möglicher Zusammenhang der genannten Faktoren sowohl mit dem Ausgangsniveau des emotionalen Erlebens wie auch mit Veränderungen im emotionalen Erleben erwartet. Wir gehen davon aus, dass Schulleistung in einem positiven Zusammenhang mit günstigerem emotionalen Erleben steht. Darüber hinaus nehmen wir an, dass der Zusammenhang mit dem Geschlecht sehr domänenspezifisch sein kann. Für Schüler*innen mit Migrationshintergrund gehen wir davon aus, dass sie ein tendenziell ungünstigeres emotionales Erleben angeben.

## Methode

### Design

Die Fragebogenstudie war in einem quantitativen Längsschnitt-Design mit fünf Messzeitpunkten angelegt. Die erste Befragung der Schüler*innen fand im Juli 2019 am Ende der Jahrgangsstufe 3 statt (Messzeitpunkt 1). Die erste Erhebung in Jahrgangsstufe 4 wurde wenige Wochen nach Beginn des Schuljahres, Ende Oktober 2019, durchgeführt (Messzeitpunkt 2). Im Januar 2020 wurden die Schüler*innen zweimal in relativ kurzem Abstand vor und nach der Herausgabe der Zwischeninformation befragt (Messzeitpunkte 3 und 4). Zum Abschluss der Studie erfolgte die Erhebung im Juni 2020 direkt nach dem Distanzlernen (Messzeitpunkt 5).

Die Schüler*innen wurden jeweils zu ihrem emotionalen Erleben im Unterricht (Freude, Angst, Langeweile) in den übertrittsrelevanten Fächern Deutsch, Mathematik und Heimat- und Sachunterricht (HSU) sowie zu ihren Freuden und Sorgen hinsichtlich des bevorstehenden Übertritts befragt. Beim letzten Messzeitpunkt wurde das emotionale Erleben (Freude, Angst, Langeweile) auf die Zeit des Distanzlernens in den oben genannten Fächern bezogen.

### Stichprobe

An der Studie nahmen insgesamt 225 Schüler*innen (116 weiblich) aus 22 Klassen an bayerischen Grundschulen teil. 120 Schüler*innen nahmen an allen fünf Messzeitpunkten, 205 Schüler*innen an mindestens vier und 219 Schüler*innen an mindestens drei Messzeitpunkten teil. Pro Klasse nahmen zwischen vier und 19 Kindern teil. Zu Beginn der Studie waren die Schüler*innen in Jahrgangsstufe 3 im Schnitt 9,53 Jahre alt (*SD* = 0,50 Jahre, *n* = 217).

Für einen Teil der Kinder (*n* = 94) liegen Elternangaben zum Migrationshintergrund vor. Von 25,5 % (*n* = 24) dieser Kinder ist mindestens ein Elternteil nicht in Deutschland geboren.

Die Note in der Zwischeninformation (Januar 2020) wurde über die Lehrer*innen für die Fächer Deutsch (*M* = 2,45, *SD* = 0,96), Mathematik (*M* = 2,46, *SD* = 1,13) und Heimat- und Sachunterricht (*M* = 2,51,* SD* = 1,08) erfasst. Insgesamt 93 Eltern gaben das Einverständnis, dass die Lehrer*innen die Noten ihrer Kinder angeben durften.

### Instrumente

Erfasst wurden die unterrichtsbezogenen Emotionen Freude (2 Items, „[FACH] macht mir Spaß.“ oder „Ich freue ich mich auf [FACH].“), Angst (2 Items, „Wenn ich an [FACH] denke, bin ich beunruhigt.“ oder „[FACH] macht mir Angst.“) und Langeweile (3 Items, z. B. „Ich finde [FACH] langweilig.“) der Schüler*innen getrennt für die Fächer Deutsch, Mathematik und Heimat- und Sachunterricht. Die Items sind einer deutschsprachigen Version des Achievement Emotions Questionnaire – Elementary School (AEQ-ES, Lichtenfeld et al. 2012) entnommen. Für den letzten Messzeitpunkt wurden die Items mit Bezug auf das Distanzlernen in den entsprechenden Fächern umformuliert (z. B. „Die [FACH]-Aufgaben haben mir Spaß gemacht.“).

Folgend auf die Einleitung „Wenn ich daran denke, dass ich nach der 4. Klasse in eine neue Schule gehen werde, …“ wurden Freuden und Sorgen in Bezug auf den bevorstehenden Übertritt mit den Schwerpunkten *soziale Integration in der neuen Klasse* (Freude: 6 Items, z. B. „… dann freue ich mich, weil ich eine Menge netter Kinder kennenlernen kann.“; Sorgen: 6 Items, z. B. „… mache ich mir Sorgen, weil ich vielleicht keine netten Freunde finde.“) und *inhaltliche Anforderungen* (Freude: 5 Items, z. B. „… dann freue ich mich, weil ich zeigen kann, was ich wirklich kann.“; Sorgen: 8 Items, z. B. „… mache ich mir Sorgen, weil ich im Unterricht vielleicht nicht mitkomme.“) mit Hilfe einer bewährten Skala von Munser-Kiefer et al. (in Vorbereitung) erhoben.

Alle Items wurden auf einer 5‑stufigen Likert-Skala von 0 (stimmt gar nicht) bis 4 (stimmt sehr) erfasst. Einen Überblick über die deskriptiven Statistiken (Mittelwert, Standardabweichung und Cronbach’s Alpha) geben Tab. 1 und 2. In Anbetracht der geringen Itemzahl weisen die Skalen für alle Konstrukte bis auf Angst angemessene Reliabilitäten auf. Die nicht ausreichende Reliabilität der Angst-Skalen ist vermutlich auf Bodeneffekte zurückzuführen. Aus inhaltlichen Gründen wurde Angst dennoch in die Verlaufsanalysen einbezogen. Da im Rahmen dieser Modelle die Ladungen beider Items auf eins gesetzt wurden, wird ausschließlich die gemeinsame Varianz beider Items berücksichtigt. Die Besonderheiten der Ergebnisse in Bezug auf die Emotion Angst werden im Ergebnisteil erläutert.

**Table 1:** 

	**Freude**								
	*Deutsch*			*Mathematik*			*HSU*		
	*M*	*SD*	*α*	*M*	*SD*	*α*	*M*	*SD*	*α*
MZP1	2,48	1,03	0,69	3,04	1,11	0,79	3,05	1,59	0,45
MZP2	2,45	1,03	0,74	2,90	1,14	0,84	2,88	1,18	0,84
MZP3	2,35	1,01	0,75	2,85	1,13	0,86	2,89	1,20	0,83
MZP4	2,40	1,11	0,81	2,75	1,17	0,85	2,90	1,12	0,79
MZP5	2,01	1,11	0,80	2,54	1,26	0,84	2,64	1,28	0,87
	**Angst**								
	*Deutsch*			*Mathematik*			*HSU*		
	*M*	*SD*	*α*	*M*	*SD*	*α*	*M*	*SD*	*α*
MZP1	0,94	0,92	0,25	0,98	1,02	0,33	1,03	0,96	0,14
MZP2	0,81	0,85	0,20	0,97	1,00	0,23	0,92	0,93	0,09
MZP3	0,70	0,77	0,18	0,86	0,96	0,33	0,92	1,03	0,25
MZP4	0,71	0,81	0,21	0,91	0,92	0,22	0,84	0,99	0,43
MZP5	0,64	0,82	0,50	0,70	0,90	0,44	0,63	0,89	0,50
	**Langeweile**								
	*Deutsch*			*Mathematik*			*HSU*		
	*M*	*SD*	*α*	*M*	*SD*	*α*	*M*	*SD*	*α*
MZP1	0,97	0,89	0,60	0,68	0,90	0,68	0,78	0,87	0,64
MZP2	0,94	0,92	0,65	0,73	0,93	0,71	0,71	0,85	0,61
MZP3	0,93	0,94	0,77	0,75	1,00	0,50	0,70	0,91	0,75
MZP4	0,87	0,86	0,69	0,72	0,85	0,71	0,65	0,86	0,76
MZP5	1,13	1,02	0,77	0,85	1,04	0,81	0,74	1,04	0,79

*N*_*MZP1*_ = 186; *N*_*MZP*__2_ = 216; *N*_*MZP3*_ = 210; *N*_*MZP4*_ = 210; *N*_*MZP5*_ = 169; Konstrukte sind auf einer fünf-stufigen Likert-Skala von 0 bis 4 erfasst

*MZP* Messzeitpunkt

**Table 2:** 

	**Vorfreude**					
	*Inhaltlich*			*Sozial*		
	*M*	*SD*	*α*	*M*	*SD*	*α*
MZP1	2,97	1,02	0,72	2,89	0,90	0,87
MZP2	2,83	1,04	0,88	2,79	1,06	0,91
MZP3	2,89	0,96	0,87	2,83	1,05	0,93
MZP4	2,85	1,07	0,91	2,82	1,15	0,91
MZP5	2,85	1,05	0,91	2,90	1,06	0,95
	**Sorgen**					
	*Inhaltlich*			*Sozial*		
	*M*	*SD*	*α*	*M*	*SD*	*α*
MZP1	1,70	1,12	0,93	1,67	1,24	0,92
MZP2	1,64	1,17	0,94	1,68	1,26	0,92
MZP3	1,47	1,10	0,94	1,51	1,20	0,92
MZP4	1,52	1,15	0,95	1,51	1,23	0,93
MZP5	1,28	1,09	0,95	1,10	1,10	0,92

*N*_*MZP1*_ = 186; *N*_*MZP*__2_ = 216; *N*_*MZP3*_ = 210; *N*_*MZP4*_ = 210; *N*_*MZP5*_ = 169; Konstrukte sind auf einer fünf-stufigen Likert-Skala von 0 bis 4 erfasst

*MZP* Messzeitpunkt

### Datenanalyse

**Figure 1:**
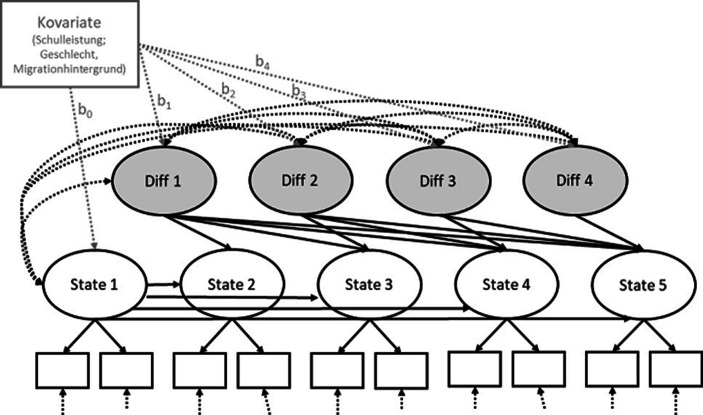

Die vertiefenden Analysen wurden mit dem Programm MPlus (Muthén und Muthén 1998–2017) durchgeführt. In Bezug auf Fragestellung I wurde für jedes der Konstrukte ein Latent Neighbour Change Modell (vgl. Abb. 1) gerechnet. Diese Modelle ermöglichen eine Quantifizierung von Veränderungen von einem Messzeitpunkt zum nächsten unter Bezugnahme des ersten Messzeitpunktes als Referenz. In allen Modellen wurde eine starke Messinvarianz der Konstrukte angenommen, das bedeutet bei Skalen mit zwei Items (Freude und Angst) wurden die Ladungen für alle Messzeitpunkte auf eins gesetzt, für alle weiteren Skalen wurden die Ladungen über die Messzeitpunkte hinweg stabil gehalten (vgl. Abb. 1). Die Fit-Indices der einzelnen Latent Neighbour Change Modelle zur Beantwortung der ersten Fragestellung sind in Tab. 3 aufgeführt. Bis auf eine Ausnahme (Sorgen vor dem Übertritt mit Schwerpunkt inhaltliche Anforderungen) wiesen alle Modelle, besonders unter Berücksichtigung der Stichprobengröße, sehr gute Modellfits auf. Fehlende Werte wurden mit Hilfe des full information maximum likelihood (FIML) geschätzt.

**Table 3:** 

Fit Indices der Latent Neighbour Change Modelle								
		Chi Square	Degrees of Freedom	*p*-value	RMSEA	CFI	TLI	SRMR
Freude	Deutsch	35,69	14	0,00	0,08	0,97	0,91	0,03
	Mathe	12,87	14	0,54	0,00	1,00	1,01	0,02
	HSU	16,44	14	0,29	0,03	1,00	0,99	0,03
Angst	Deutsch	23,04	14	0,06	0,05	0,97	0,90	0,04
	Mathe	23,05	14	0,06	0,05	0,97	0,90	0,04
	HSU	13,04	14	0,52	0,00	1,00	1,01	0,03
Langeweile	Deutsch	74,89	66	0,21	0,02	0,99	0,99	0,05
	Mathe	79,49	66	0,12	0,03	0,98	0,97	0,05
	HSU	100,70	66	0,00	0,05	0,95	0,92	0,06
Übertrittsfreude	Sozial	632,04	375	0,00	0,06	0,93	0,91	0,06
	Inhaltlich	428,55	247	0,00	0,06	0,92	0,91	0,07
Übertrittssorgen	Sozial	505,76	375	0,00	0,04	0,96	0,96	0,05
	Inhaltlich	1329,58	706	0,00	0,06	0,88	0,87	0,07

*N*_*MZP1*_ = 186; *N*_*MZP*__2_ = 216; *N*_*MZP3*_ = 210; *N*_*MZP4*_ = 210; *N*_*MZP5*_ = 169; Konstrukte sind auf einer fünf-stufigen Likert-Skala von 0 bis 4 erfasst. Modelle entsprechen der Darstellung in Abb. 1

Zur Beantwortung der Fragestellung II wurden Kovariaten in die Modelle einbezogen (vgl. Abb. 1). Die Note in der Zwischeninformation wurde vor dem Einbezug in die Modelle am Stichprobenmittelwert zentriert. In die Modelle der fachspezifischen Emotionen wurden ausschließlich die Noten der entsprechenden Fächer in die Modelle aufgenommen. Für die Modelle zu Freuden und Sorgen in Bezug auf den Übertritt wurde der zentrierte mittlere Wert der drei Noten aufgenommen. Noten wurden auf der in Deutschland üblichen Skala von 1 bis 6 erfasst, ein höherer Wert geht somit mit einer schlechteren Leistung einher. Geschlecht (0 = männlich; 1 = weiblich) und Migrationshintergrund (0 = „kein Migrationshintergrund“; 1 = „mindestens ein Elternteil ist nicht in Deutschland geboren“) wurden über dichotome Items erfasst. Aufgrund der deutlich reduzierten Stichprobenanzahl für die Konstrukte Note und Migrationshintergrund wurde für jedes Konstrukt ein einzelnes Modell analysiert.

Die Mehrebenenstruktur der Daten wurde innerhalb der Analysen berücksichtigt (type is complex), dazu wurde der MLR Schätzer genutzt.

## Ergebnisse

Die deskriptiven Befunde (vgl. Tab. 1 und 2) zeigen, dass auf den ersten Blick positive Emotionen insgesamt deutlich ausgeprägter zu sein scheinen als negative Emotionen. Sowohl Angst als auch Langweile weisen in allen Fächern leichte Bodeneffekte auf.

### Emotionales Erleben in Jahrgangsstufe 4 – im Covid-19 Jahr 2020

Die Ergebnisse der einzelnen Modelle sind in Tab. 4 und 5 aufgeführt. Für die Interpretation ist an dieser Stelle wichtig, die Varianzen ebenfalls zu betrachten. Auch bei einem latenten Mittelwert, der sich nicht von Null unterscheidet, kommt es, sofern sich die Varianz von Null unterscheidet, zu Veränderungen im emotionalen Erleben einzelner Schüler*innen.

**Table 4:** 

	**Freude**											
	*Deutsch*				*Mathe*				*HSU*			
	*M*	*p*	*Var*	*p*	*M*	*p*	*Var*	*p*	*M*	*p*	*Var*	*p*
MZP1	2,61	0,00	0,74	0,00	3,18	0,00	0,94	0,00	3,07	0,00	0,97	0,00
Differenz 1–2	−0,04	0,52	0,09	0,27	−0,12	0,09	0,31	0,03	−0,09	0,42	0,59	0,01
Differenz 2–3	−0,09	0,11	0,25	0,01	−0,06	0,37	0,48	0,00	0,02	0,84	0,57	0,00
Differenz 3–4	0,04	0,50	0,07	0,31	−0,12	0,12	0,32	0,06	0,00	1,00	0,20	0,19
Differenz 4–5	−0,34	0,00	0,77	0,00	−0,15	0,05	0,75	0,00	−0,28	0,02	0,92	0,00
	**Angst**											
	*Deutsch*				*Mathe*				*HSU*			
	*M*	*p*	*Var*	*p*	*M*	*p*	*Var*	*p*	*M*	*p*	*Var*	*p*
MZP1	1,22	0,00	0,20	0,02	1,29	0,00	0,32	0,03	1,33	0,00	0,05	0,63
Differenz 1–2	−0,11	0,15	0,01	0,95	0,04	0,64	0,23	0,16	−0,10	0,19	0,00	–
Differenz 2–3	−0,04	0,40	0,09	0,43	−0,04	0,52	0,35	0,09	0,00	0,99	0,02	0,89
Differenz 3–4	0,02	0,64	−0,09	0,22	0,05	0,37	0,00	0,99	−0,02	0,56	0,16	0,10
Differenz 4–5	−0,10	0,06	−0,04	0,75	−0,18	0,00	0,15	0,31	−0,22	0,00	0,26	0,01
	**Langeweile**											
	*Deutsch*				*Mathe*				*HSU*			
	*M*	*p*	*Var*	*p*	*M*	*p*	*Var*	*p*	*M*	*p*	*Var*	*p*
MZP1	1,04	0,00	0,78	0,00	0,62	0,00	0,71	0,00	0,79	0,00	0,68	0,00
Differenz 1–2	−0,02	0,79	0,42	0,01	0,07	0,32	0,56	0,01	−0,09	0,32	0,33	0,03
Differenz 2–3	0,00	1,00	0,51	0,01	−0,04	0,30	0,43	0,00	−0,04	0,72	0,74	0,00
Differenz 3–4	−0,06	0,30	0,46	0,00	0,03	0,60	0,25	0,01	0,00	0,99	0,64	0,00
Differenz 4–5	0,26	0,00	0,84	0,00	0,16	0,10	0,99	0,00	0,07	0,46	1,08	0,00

*N*_*MZP1*_ = 186; *N*_*MZP*__2_ = 216; *N*_*MZP3*_ = 210; *N*_*MZP4*_ = 210; *N*_*MZP5*_ = 169; Dargestellt sind die Mittelwerte und Varianzen der latenten Variablen der Latent Neighbour Change Basismodelle. Mittelwert und Varianz zum MZP 1 stellen somit den Ausgangswert dar (vergleiche State 1 in Abb. 1), Differenz 1–2 bezeichnet die Werte der latenten Differenzvariablen zwischen dem ersten und dem zweiten Messzeitpunkt und somit die Veränderung während der Sommerferien, Differenz 2–3 die Veränderung von Beginn des Schuljahres bis kurz vor der Zwischeninformation, Differenz 3–4 die Veränderung zwischen den Zeiträumen kurz vor und kurz nach der Zwischeninformation und Differenz 4–5 die Veränderung während des Distanzlernens

*MZP* Messzeitpunkt

**Table 5:** 

	**Übertrittsfreude**							
	*Inhaltlich*				*Sozial*			
	*M*	*p*	*Var*	*p*	*M*	*p*	*Var*	*p*
MZP1	3,09	0,00	0,68	0,00	2,83	0,00	0,66	0,00
Differenz 1–2	−0,13	0,10	0,58	0,00	−0,07	0,39	0,64	0,00
Differenz 2–3	0,05	0,48	0,58	0,00	0,04	0,46	0,49	0,00
Differenz 3–4	−0,04	0,50	0,35	0,00	−0,03	0,57	0,34	0,00
Differenz 4–5	−0,01	0,93	1,11	0,00	0,04	0,69	0,95	0,00
	**Sorgen vor dem Übertritt**							
	*Inhaltlich*				*Sozial*			
	*M*	*p*	*Var*	*p*	*M*	*p*	*Var*	*p*
MZP1	1,41	0,00	1,06	0,00	1,85	0,00	1,60	0,00
Differenz 1–2	−0,03	0,67	0,68	0,00	0,03	0,66	0,77	0,00
Differenz 2–3	−0,17	0,00	0,49	0,00	−0,13	0,04	0,88	0,00
Differenz 3–4	0,06	0,25	0,46	0,00	−0,07	0,39	0,47	0,00
Differenz 4–5	−0,23	0,01	0,94	0,00	−0,37	0,00	1,29	0,00

*N*_*MZP1*_ = 186; *N*_*MZP*__2_ = 216; *N*_*MZP3*_ = 210; *N*_*MZP4*_ = 210; *N*_*MZP5*_ = 169; Dargestellt sind die Mittelwerte und Varianzen der latenten Variablen der Latent Neighbour Change Basismodelle. Mittelwert und Varianz zum MZP 1 stellen somit den Ausgangswert dar (vergleiche State 1 in Abb. 1), Differenz 1–2 bezeichnet die Werte der latenten Differenzvariablen zwischen dem ersten und dem zweiten Messzeitpunkt und somit die Veränderung während der Sommerferien, Differenz 2–3 die Veränderung von Beginn des Schuljahres bis kurz vor der Zwischeninformation, Differenz 3–4 die Veränderung zwischen den Zeiträumen kurz vor und kurz nach der Zwischeninformation und Differenz 4–5 die Veränderung während des Distanzlernens

*MZP* Messzeitpunkt

In allen drei Fächern ist die Freude zum Ende der dritten Jahrgangsstufe auf einem relativ hohen Ausgangsniveau und sinkt erst und ausschließlich unter der Bedingung der Schulschließungen signifikant ab. Im Fach Deutsch ist die Varianz dieses Differenzwertes die einzig signifikante von Null unterschiedliche Varianz. In den Fächern Mathematik und HSU weisen darüber hinaus auch die Differenzwerte, die den Wechsel von Jahrgangsstufe 3 und 4 sowie die Entwicklung zwischen Oktober und Anfang Januar markieren, eine signifikante Varianz auf.

In allen drei Fächern weist die Emotion Angst ein relativ niedriges Ausgangsniveau zum Ende der dritten Jahrgangsstufe auf. Die einzig signifikante Veränderung ist eine nochmalige Verringerung der fachbezogenen Angst während des Distanzlernens in Mathematik und HSU. Allerdings weist bis auf den latenten Differenzwert zu diesem letzten Messzeitpunkt im Fach HSU kein Differenzwert eine signifikante Varianz auf. Auf Basis der nicht von Null unterschiedlichen Varianzen wird auf einen Einbezug von Kovariaten für diese Emotion verzichtet.

Mit Bezug auf das Fach Deutsch steigt das Erleben von Langeweile aufgrund des Distanzlernens signifikant an, für die Fächer Mathematik und HSU ist kein signifikanter Anstieg zu verzeichnen. Die Varianzen aller latenten Variablen des Modells werden signifikant.

Die Vorfreude auf den bevorstehenden Übertritt sowohl in Bezug auf die inhaltlichen als auch in Bezug auf die sozialen Herausforderungen ist ebenfalls auf einem relativ stabilen hohen Niveau. Bei nicht signifikanten Veränderungswerten weisen die latenten Variablen alle eine signifikante Varianz auf.

Die Sorgen vor dem bevorstehenden Übertritt sind sowohl in Bezug auf inhaltliche als auch in Bezug auf soziale Herausforderungen zunächst auf einem relativ niedrigen Niveau und sinken zwischen Oktober (MZP 2) und Januar (MZP 3) und dann nochmals während des Distanzlernens signifikant ab. Auch für diese Konstrukte sind alle Varianzen signifikant von Null verschieden.

### Emotionales Erleben, Schulleistung, Geschlecht und Migrationshintergrund

Im Sinne der Übersichtlichkeit werden im Folgenden nur signifikante Ergebnisse berichtet. Zu jedem der Basismodelle zu Freude (3), Langeweile (3), übertrittsbezogene Freude (2) und Sorgen (2) wurden je drei zusätzliche Modelle unter Einbezug der Kovariaten Schulleistung, Geschlecht und Migrationshintergrund, insgesamt also 30 Modelle, analysiert.

#### Freude im Zusammenhang mit Schulleistung, Geschlecht und Migrationshintergrund

Die Note in der Zwischeninformation hängt signifikant negativ mit dem Ausgangsniveau der Freude im Fach Deutsch (*b*_*0*_ = −0,34; *p* < 0,01), im Fach Mathematik (*b*_*0*_ = −0,37; *p* < 0,01) und in HSU (*b*_*0*_ = −0,30; *p* = 0,01) zusammen. Leistungsschwächere Schüler*innen erlebten somit weniger fachbezogene Freude. Allerdings sinkt für leistungsschwächere Schüler*innen im Fach HSU die Freude während des Distanzlernens weniger stark ab (*b*_*4*_ = 0,23; *p* = 0,03). Während Mädchen mehr Freude im Fach Deutsch erlebten als Jungen (*b*_*0*_ = 0,46; *p* < 0,01) gibt es für das Fach Mathematik umgekehrte Effekte (*b*_*0*_ = −0,33;* p* = 0,04).

#### Langeweile im Zusammenhang mit Schulleitung, Geschlecht und Migrationshintergrund

Die Note im entsprechenden Fach hängt mit dem Erleben von Langeweile sowohl in Deutsch, als auch in HSU zusammen. Leistungsschwächere Schüler*innen berichteten mehr Langeweile (Deutsch: *b*_*0*_ = 0,27; *p* = 0,02; HSU: *b*_*0*_ = 0,21; *p* < 0,01). Es zeigt sich ein negativer Zusammenhang mit der Veränderung der Langeweile während des Distanzlernens (Deutsch: *b*_*4*_ = −0,27; *p* = 0,049; HSU *b*_*4*_ = −0,33; *p* = 0,02). Dieses Ergebnis legt die Vermutung nahe, dass leistungsschwächere Schüler*innen sich zwar tendenziell verstärkt langweilen, diese Langeweile aber während der Schulschließungen nicht noch intensiver wurde. Mädchen erlebten in Bezug auf das Fach Deutsch weniger Langeweile als Jungen (*b*_*0*_ = −0,37; *p* = 0,03). Auch auf den Differenzwert zum letzten Messzeitpunkt gibt es einen negativen Zusammenhang mit dem Geschlecht (*b*_*4*_ = −0,28; *p* = 0,03). Dies bedeutet, dass für Mädchen während des Distanzlernens die Langeweile in Bezug auf das Fach Deutsch im Schnitt weniger stark ansteigt als für Jungen. Für die Fächer Mathematik und HSU zeigen sich keine Effekte. Im Fach Deutsch zeigt sich ein positiver Zusammenhang zwischen dem Migrationshintergrund und der Veränderung der Langeweile während der Sommerferien (*b*_*1*_ = 0,31; *p* = 0,01) und ein negativer Zusammenhang zur Veränderung während des Distanzlernens (*b*_*4*_ = −0,40;* p* = 0,04). In Bezug auf das Fach Mathematik erlebten Schüler*innen mit Migrationshintergrund im Ausgangsniveau weniger Langeweile als Schüler*innen ohne Migrationshintergrund (*b*_*0*_ = −0,42;* p* = 0,01).

#### Übertrittsfreude im Zusammenhang mit Schulleistung, Geschlecht und Migrationshintergrund

Der Mittelwert der Noten in der Zwischeninformation über die Fächer Deutsch, Mathematik und HSU steht in keinem Zusammenhang mit dem Ausgangsniveau der Vorfreude mit Bezug auf inhaltliche und soziale Herausforderungen des Übertritts. Im Zeitraum von Oktober (MZP 2) bis Januar (MZP 3) ist die Note jedoch mit einem marginal signifikanten Anstieg in der Vorfreude auf inhaltliche Herausforderungen (*b*_*2*_ = 0,10; *p* = 0,07) und mit einem signifikanten Anstieg in der Vorfreude auf soziale Herausforderungen (*b*_*2*_ = 0,14; *p* = 0,02) assoziiert. Dies bedeutet, dass bei Schüler*innen, die schlechtere Schulleistungen in der Zwischeninformation erhalten, in diesem Zeitraum die Freude zunimmt (Anmerkung: höhere Notenwertung geht mit schlechterer Leistung einher).

Obwohl sich für die inhaltliche Vorfreude im Ausgangsmodell keine signifikanten Veränderungen im Laufe der Studiendauer zeigen, wird die Veränderung der inhaltlichen Vorfreude signifikant, wenn man das Geschlecht einbezieht. In diesem Fall zeigt sich ein signifikantes Absinken der Vorfreude während der Sommerferien (*Differenz 1–2* = −0,25; *p* = 0,02) mit einem Einfluss des Geschlechts (*b*_*1*_ = 0,25; *p* = 0,03). Dieses Ergebnis legt die Interpretation nahe, dass während der Sommerferien die Vorfreude der Jungen absinkt, während die Freude auf die bevorstehenden Herausforderungen bei den Mädchen stabil bleibt.

#### Übertrittssorgen im Zusammenhang mit Schulleistung, Geschlecht und Migrationshintergrund

Die Sorgen vor inhaltlichen Herausforderungen des Übertritts stehen ausschließlich mit der Note in der Zwischeninformation in Zusammenhang. Leistungsschwächere Schüler*innen machten sich bereits zum Ende der Jahrgangsstufe 3 in der Tendenz sowohl mehr inhaltliche Sorgen (*b*_*0*_ = 0,33; *p* < 0,01) als auch mehr soziale Sorgen (*b*_*0*_ = 0,28; *p* < 0,01). Es zeigt sich auch eine marginal signifikante Verstärkung dieses Effekts zum Ende der Jahrgangsstufe 4 während des Distanzlernens (*b*_*4*_ = 0,27; *p* = 0,056). Schüler*innen mit Migrationshintergrund zeigen ein höheres Ausgangsniveau (*b*_*0*_ = 0,88; *p* < 0,01) in Sorgen vor sozialen Herausforderungen.

## Diskussion und Limitationen

Im Folgenden wird die Entwicklung des emotionalen Erlebens vom Ende der 3. Jahrgangsstufe bis zum Ende der 4. Jahrgangsstufe sowie der Einfluss von Schulleistung und weiteren individuellen Faktoren diskutiert.

### Die Entwicklung des emotionalen Erlebens in Jahrgangsstufe 4 – im Covid-19 Jahr 2020

Zusammenfassend zeigt sich, dass im Schuljahr 2019/2020 das Distanzlernen in Bezug auf die fachbezogenen Emotionen Freude, Angst und Langeweile eine bedeutende Rolle gespielt hat. Schüler*innen erlebten während dieser Zeit in allen Fächern weniger Freude und mehr Langeweile im Fach Deutsch. Soweit man die Ergebnisse zu Angst vorsichtig interpretieren kann, erlebten sie jedoch auch weniger Angst in Mathematik und HSU. Diese Effekte stehen in Einklang mit der Annahme, dass insbesondere die soziale Situation einen großen Einfluss auf das Erleben von Lern- und Leistungsemotionen hat. Schüler*innen erleben beim Distanzlernen weniger soziale Bestärkung und somit auch weniger Valenz, was zu einer Verringerung der Freude und zu einem Anstieg von Langeweile führen kann. Die fehlende soziale Konkurrenz kann jedoch auch die Angst abmildern.

Im Gegensatz zum Distanzlernen stehen weitere Ereignisse des Schuljahres, wie beispielsweise der Erhalt der Zwischeninformation, in keinem Zusammenhang mit Veränderungen im emotionalen Erleben der Schüler*innen. Dies weist darauf hin, dass sich der bevorstehende Übertritt und die damit verbundenen Herausforderungen nicht notwendigerweise und gleichermaßen auf das aktuelle emotionale Erleben von allen Schüler*innen auswirken. Es bedeutet jedoch nicht, dass sich die mit dem Übertritt verbundenen Herausforderungen nicht auf die allgemeine Wahrnehmung einzelner Schüler*innen auswirken und als Stressor wirken können. Hierfür sind die Varianzen der Veränderungsvariablen ein Indiz. Zukünftige Experience Sampling Studien in Kombination mit Fragebogenuntersuchungen könnten weitere Hinweise auf intraindividuelle Veränderungen geben.

Einen Hinweis darauf, dass der Übertritt und die damit verbundenen Ereignisse dennoch eine Rolle für übergreifende Appraisals spielen, gibt die Veränderung in übertrittsbezogenen Sorgen während der ersten Hälfte des vierten Schuljahres. Dies mag für die Tatsache sprechen, dass sich für die meisten Schüler*innen bereits während des ersten Schulhalbjahres eine Schullaufbahnentscheidung herauskristallisiert. Ob ein weiteres Absinken der übertrittsbezogenen Sorgen während des Distanzlernens auf die Covid-19-Situation oder auf das Schuljahresende zurückzuführen ist, bleibt ungeklärt.

In Summe belegen das überwiegend hohe Niveau positiver und das niedrige Niveau negativer Emotionen sowie die stabil bleibende übertrittsbezogene Vorfreude, dass das emotionale Erleben von Schüler*innen im Schnitt ein günstiges Muster aufweist. Die Ergebnisse untermauern die Annahmen, dass Schüler*innen den Übertritt eher als positive Herausforderung denn als Stressor betrachten (vgl. Kurtz et al. 2010). Die meist signifikanten Varianzen belegen jedoch auch, dass dies für einzelne Schüler*innen nicht gelten muss. Der Frage, inwiefern individuelle Faktoren eine Rolle spielen, kommt also eine wichtige Bedeutung bei. Zugleich ist limitierend anzumerken, dass die Stichprobe nicht repräsentativ ist und einer hohen Selbstselektion unterlag, so dass die Befunde auch durch die teilselektierte Stichprobe oder einen gewissen Dropout, insbesondere zum letzten Messzeitpunkt, beeinflusst sein könnten.

### Schulleistung, Geschlecht und Migrationshintergrund

Der Interpretation zum Zusammenhang mit den Kovariaten Schulleistung, Geschlecht und Migrationshintergrund ist ebenfalls vorwegzuschicken, dass für Schulleistung und Migrationshintergrund nur jeweils von einem Teil der Schüler*innen die entsprechenden Informationen vorlagen.

Die vorliegenden Befunde zum Zusammenhang zwischen dem emotionalen Erleben von Grundschüler*innen und Schulleistung, Geschlecht oder Migrationshintergrund sind insgesamt sehr heterogen. Schulleistung steht mit fast allen emotionsbezogenen Konstrukten in Zusammenhang. Diese Befunde stehen somit in Einklang mit bisherigen empirischen Befunden, die von Wechselwirkungen zwischen emotionalem Erleben und Schulleistung ausgehen (vgl. z. B. Camacho-Morles et al. 2021, Helmke 1993; Lichtenfeld et al. 2012; Pekrun et al. 2017; Raccanello et al. 2019). Ein interessantes Ergebnis ist, dass für leistungsschwächere Schüler*innen die Langeweile während des Distanzlernens weniger anstieg als für leistungsstärkere Schüler*innen. Dies ist zum einen natürlich intuitiv, berücksichtigt man das niedrigere Ausgangsniveau im Erleben von Langeweile der leistungsstärkeren Schüler*innen, mag aber auch ein vorsichtiges Indiz darauf sein, dass im Erleben von Langeweile der verstärkende Effekt des Verlusts von sozialen Strukturen durch mehr Autonomie und eine geringere Überforderung während des Distanzlernens abgefedert wurde (vgl. hierzu auch Befunde zur Kontroll-Wert Theorie von Pekrun et al. 2006). Dass bereits vor dem Zeitpunkt der Zwischeninformation bei leistungsschwächeren Schüler*innen die Vorfreude auf den Übertritt im sozialen Kontext zunimmt, könnte daran liegen, dass für sie der Druck hinsichtlich des Übertrittszeugnisses vielleicht schon nachgelassen hat und eine sichere Entscheidung getroffen ist, während Schüler*innen, bei denen noch verschiedene Übertrittsmöglichkeiten im Raum stehen, mehr Druck und damit eine geringere Vorfreude, gerade auch auf die sozialen Herausforderungen, erleben. Wichtig an dieser Stelle ist nochmals anzumerken, dass wir keine Schlüsse über Kausalitäten ziehen können.

Geschlecht und Migrationshintergrund stehen im Zusammenhang mit dem emotionalen Erleben. Hier zeigen sich auch differentielle Effekte der Gruppen (z. B. Vorfreude der Jungen). Da das Vorgehen zum Zusammenhang mit den Kovariaten durchaus auch explorativ war und sehr viele Einzelanalysen durchgeführt wurden, können die Ergebnisse jedoch nur als erste Hinweise interpretiert werden und müssen in weiteren Studien zunächst repliziert und vertieft werden.

## Ausblick

Insgesamt zeigen die Ergebnisse der vorliegenden Studie, dass Schüler*innen der Grundschule auch in der 4. Jahrgangstufe im Schnitt ein eher günstiges emotionales Muster aufweisen. Dieses kann jedoch für bestimmte Schüler*innen und unter bestimmten Voraussetzungen durchaus bedroht werden. Eine verstärkte Berücksichtigung weiterer Situationen, wie z. B. von Hausaufgaben oder Prüfungssituationen, könnte einen vertiefenden Einblick in das emotionale Erleben der Schüler*innen geben. Der deutliche Zusammenhang des emotionalen Erlebens mit der Schulleistung belegt außerdem, dass beides nicht losgelöst voneinander zu betrachten ist. Das Distanzlernen hat einen merklichen und nachweisbaren Einfluss auf das emotionale Erleben der Schüler*innen gehabt. Während jedoch möglichen durch das Distanzlernen bedingten Lernrückständen aktuell Rechnung getragen werden soll, ist das emotionale und auch das motivationale Erleben der Schüler*innen und der Einfluss des Distanzlernens auf ebendiese bisher kaum in den Fokus von schulbezogener Politik und Forschung gerückt worden. Weitere Studien, insbesondere praxisnahe Interventionsstudien und eine enge Rückkoppelung von Forschung und Praxis, sollten dem emotionalen und motivationalen Erleben in Zukunft verstärkt Rechnung tragen.
